# Resveratrol Relieves Hepatic Steatosis and Enhances the Effects of Atorvastatin in a Mouse Model of NAFLD by Regulating the Renin‐Angiotensin System, Oxidative Stress, and Inflammation

**DOI:** 10.1002/fsn3.70073

**Published:** 2025-03-04

**Authors:** Esmaeel Babaeenezhad, Navid Farahmandian, Mohammadjavad Sotoudeheian, Omid Dezfoulian, Elaheh Askari, Niloofar Taghipour, Sahar Yarahmadi

**Affiliations:** ^1^ Nutritional Health Research Center Lorestan University of Medical Sciences Khorramabad Iran; ^2^ Department of Tissue Engineering & Regenerative Medicine, Faculty of Advanced Technologies in Medicine Iran University of Medical Sciences Tehran Iran; ^3^ Physiology Research Center, Faculty of Medicine Iran University of Medical Sciences Tehran Iran; ^4^ Department of Pathobiology, School of Veterinary Medicine Lorestan University Khorramabad Iran; ^5^ Cellular and Molecular Biology Research Center Shahid Beheshti University of Medical Sciences Tehran Iran; ^6^ Department of Tissue Engineering and Applied Cell Sciences, School of Advanced Technologies in Medicine Shahid Beheshti University of Medical Sciences Tehran Iran

**Keywords:** atorvastatin, inflammation, nonalcoholic fatty liver disease, oxidative stress, renin‐angiotensin system, resveratrol

## Abstract

The classical renin‐angiotensin system (RAS) axis is implicated in NAFLD development by promoting oxidative stress and inflammation, whereas the nonclassical axis antagonizes its effects. In the present study, we evaluated the effects of resveratrol (RSV), a polyphenol antioxidant, alone and in combination with atorvastatin (AT) on the RAS axes in NAFLD mice. Male C57/BL6 mice were fed a normal diet (control group) or a high‐fat diet (HFD) for 12 weeks to induce NAFLD. Afterwards, the animals received AT (20 mg/kg), RSV (100 mg/kg/day), and AT + RSV (20 and 100 mg/kg/day) by oral gavage for 4 weeks. NAFLD animals exhibited swollen hepatocytes with numerous fat‐containing vacuoles. Serum alanine aminotransferase (ALT) and aspartate aminotransferase (AST) activities were increased in NAFLD mice. Additionally, HFD mice exhibited dyslipidemia, as manifested by increased cholesterol (Chol), triglyceride (TG), and low‐density lipoprotein cholesterol (LDL‐C), and decreased high‐density lipoprotein cholesterol (HDL‐C). HFD significantly increased oxidative stress, as manifested by high levels of malondialdehyde and low paraoxonase 1 activity. Additionally, NAFLD mice showed significantly increased IL‐1β, IL‐6, and TNF‐α expression and reduced IL‐10 expression. An imbalance among RAS axes was evident as high expression levels of angiotensinogen, renin, and type 1 angiotensin receptor and reduced expression levels of angiotensin‐converting enzyme 2 and angiotensin 1–7. RSV ameliorated these changes in NAFLD mice, which were comparable with the beneficial effects of AT. Interestingly, the ameliorative effects of AT increased considerably when it was administered in combination with RSV. Overall, our findings indicate that RSV attenuates HFD‐induced NAFLD in mice, particularly when co‐administered with AT, at least by regulating the RAS axes, oxidative stress, and inflammation.

## Graphical abstract

In mice with non‐alcoholic fatty liver disease (NAFLD), the classical renin‐angiotensin (RAS) axis was activated, leading to oxidative stress, inflammation, and histopathological changes in the liver. In contrast, the new RAS axis was inactivated, as manifested by the downregulation of angiotensin‐converting enzyme 2 (ACE2) and angiotensin 1‐7 (Ang1‐7). Administration of resveratrol and atorvastatin considerably ameliorated NAFLD in mice by modulating new and classical RAS axes.

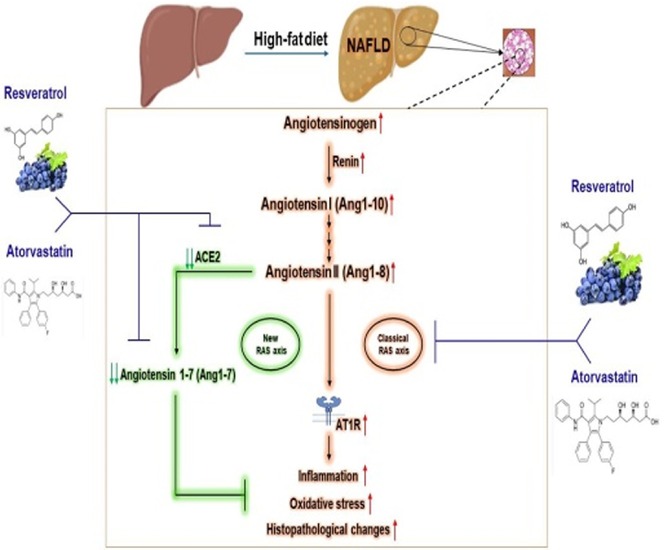

## 1 Introduction

Nonalcoholic fatty liver disease (NAFLD) is increasingly being recognized as a serious public health concern worldwide (Silva et al. 2017). It is characterized by the accumulation of fat, especially triglycerides (TG), in the liver, starting from simple steatosis and continuing to nonalcoholic steatohepatitis (NASH), fibrosis, and cirrhosis (Drescher et al. 2019). NAFLD is well known as the most frequent chronic liver disease, with a frequency of approximately 20%–30% in the general population worldwide (Gallego‐Durán and Romero‐Gómez 2015). Although the pathogenesis of NAFLD is not completely known, a number of variables such as oxidative stress, insulin resistance, lipid metabolism changes, and mitochondrial dysfunction have been reported (Rolo et al. 2012). Oxidative stress has a critical role in hepatic injury in NAFLD (Delli Bovi et al. 2021). Increased free fatty acid oxidation in hepatocytes leads to the overgeneration of reactive oxygen species (ROS) in peroxisomes, mitochondria, and microsomes, triggering lipid peroxidation and cell death. Interestingly, ROS stimulates Kupffer cells to release inflammatory cytokines, which in turn can activate stellate cells. These cells increase liver fibrosis and recruit neutrophils by producing pro‐inflammatory mediators (Rolo et al. 2012). Although various efforts have been conducted to manage NAFLD, a definitive remedy has not yet been identified.

The renin‐angiotensin system (RAS) is an intricate network of chemicals, enzymes, and receptors that contribute to the formation of metabolic syndromes and liver diseases, such as NAFLD. In addition to the systemic RAS, certain organs, including the liver, pancreas, and heart, possess a local RAS that exhibits diverse physiological effects at the cellular level through autocrine, endocrine, and paracrine mechanisms (Kon et al. 1998; Georgescu 2008; Neo et al. 2010; Santos et al. 2018). There are two classical and new RAS axes, both locally and in the bloodstream. The classical RAS axis is compromised by angiotensin‐converting enzyme (ACE), angiotensin II (Ang II), and type 1 angiotensin receptor (AT1R), whereas the new RAS axis includes angiotensin‐converting enzyme 2 (ACE2), angiotensin 1–7 (Ang1‐7), and the Mas receptor (Santos et al. 2018). ACE2 is a homologous ACE enzyme that converts Ang II to Ang1‐7 and regulates its levels. Ang II activates AT1R, leading to oxidative stress, inflammation, and fibrosis, whereas Ang1‐7 activates the Mas receptor and predominantly inhibits the detrimental effects of Ang II (Yang et al. 2020).

There is some evidence regarding the role of RAS axes in the pathogenesis of chronic liver diseases, such as hepatic fibrosis and chronic hepatitis (Yang et al. 2020). Interestingly, an imbalance between RAS axes has been reported in NAFLD. The classical RAS axis is dramatically activated, decreasing fatty acid oxidation and increasing hepatic steatosis, insulin resistance, inflammation, oxidative stress, and fibrosis (Jayasooriya et al. 2008; Wei et al. 2008; Yang et al. 2020). Blocking this axis is believed to relieve NAFLD in both patients and animal models (Yokohama et al. 2004; Yang et al. 2020). In contrast, suppression of the ACE2/Ang1‐7 axis aggravates NAFLD by upregulating lipogenic genes and downregulating fatty acid oxidation‐related genes (Cao et al. 2019).

Resveratrol (RSV), 3,5,4‐trihydroxystilbene, is a polyphenolic compound that is abundant in grapes and berries (Castro et al. 2020). RSV is well‐known for its health‐promoting effects, particularly in metabolic diseases such as diabetes mellitus, obesity, and hyperlipidemia (Mozafari et al. 2015; Sharma et al. 2017). It is believed to be less toxic and have fewer side effects than synthetic medications (Miyazaki et al. 2008). RSV also exhibits cardioprotective (Dong et al. 2014), neuroprotective (Castro et al. 2020), and vasculoprotective (Breuss et al. 2019) properties. RSV has also been shown to exert hepatoprotective effects in various liver injury models in rats (Izzo et al. 2021). RSV ameliorates cholestasis‐induced liver fibrosis in rats by regulating the TGFβ1/Smad3/miR‐21 axis (ShamsEldeen et al. 2021). RSV has been demonstrated to decrease liver oxidative stress and histopathological changes in rats with liver ischemia/reperfusion (Gedik et al. 2008). Totonchi et al. determined that RSV modulates the unfolded protein response pathway after liver ischemic injury in mice, consequently increasing liver cell longevity (Totonchi et al. 2022). These beneficial effects originate from its pharmacological activities, such as antioxidant, anti‐inflammatory (Feng et al. 2020), anti‐apoptotic (Zhang et al. 2019), antifibrotic (Rahal et al. 2012), and hypolipidemic (Sharma et al. 2017) effects. The beneficial effects of RSV can be related to its ability to regulate RAS axes in several pathological states by suppressing the ACE/Ang II/AT1R axis and activating the ACE2/Ang1‐7 axis (Jang et al. 2018). Interestingly, RSV exhibited anti‐obesity and hypolipidemic effects in ovariectomized rats. It also ameliorates insulin resistance and glucose metabolism in diabetic rats (Szkudelska et al. 2019, 2021). Considering these beneficial effects, we hypothesized that RSV may ameliorate NAFLD in rats, particularly by modulating the RAS axes, oxidative stress, and inflammation. In the current study, we evaluated the potential role of RSV in regulating the classical and nonclassical RAS axes and alleviating liver histopathological changes, oxidative stress, and inflammation in mice with NAFLD. Furthermore, we sought to determine whether RSV could enhance the effects of atorvastatin (AT) on NAFLD.

## 2 Materials and Methods

### 2.1 Animals and Experimental Design

This study involving animal experiments was conducted in accordance with the ethical guidelines and regulations set by the Ministry of Health of Iran (ethical code: IR.SBMU.AEC.1402.058) and the Department of Laboratory Animal Care of the Shahid Beheshti University of Medical Sciences. All animal experiments were conducted in accordance with the principles of the Declaration of Helsinki. The experimental models used in this study consisted of 30 male C57/BL6 mice, which were acquired from the Animal Laboratory Center of Iran University of Medical Sciences. Mice with an average weight ranging from 15 to 20 g were kept in a temperature‐controlled environment maintained at 23°C ± 2°C, with a humidity level of 55% ± 5%. Mice were exposed to a 12‐h light and 12‐h dark cycle and had unrestricted access to food and water.

After an acclimation period of 7 days, the animals were divided into five groups of six mice each. The mice in group I served as healthy controls (control group) on a standard chow diet, and the remaining mice were fed a high‐fat diet (HFD) containing 60% fat for 12 weeks to develop NAFLD and were equally placed in groups II–V (Yarahmadi et al. 2023). After 12 weeks, the animals were treated with AT, RSV, or RSV + AT for 4 weeks (until 16th week) as follows:

- Group II (NAFLD group): animals received normal saline via oral gavage.
- Group III (NAFLD + AT group): mice were treated with AT (20 mg/kg) via oral gavage.
- Group IV (NAFLD + RSV group): animals received 100 mg/kg/day RSV via oral gavage.
- Group V (NAFLD + AT + RSV group): mice were administered AT (20 mg/kg/day) and RSV (100 mg/kg/day) by oral gavage.

The doses chosen for AT and RSV were based on previous studies that reported their protective effects with no toxicity (Heebøll et al. 2014; Gao et al. 2017).

### 2.2 Blood and Tissue Collection

Following 4 weeks of treatment, the mice were profoundly anesthetized using an intraperitoneal injection of ketamine (10 mg/kg) and xylazine (100 mg/kg) (Abou‐Abbass et al. 2016). The animals were euthanized immediately by decapitation under deep anesthesia, and cardiac blood samples were collected to isolate the serum for biochemical analysis. Serum samples were separated from blood samples by centrifuging them at 3000 rpm (15 min); the serum samples were then stored at −20°C until needed. Liver tissue samples were cryopreserved at −70°C immediately after rapid transfer to liquid nitrogen. Prior to biochemical analysis, the hepatic tissues were homogenized in cold PBS (pH 7.4). Homogenized samples were centrifuged (18,000 *g*) for 15 min at 4°C to collect the supernatant. Some parts of the liver tissue were preserved in 10% neutral buffered formalin for the evaluation of histopathological changes.

### 2.3 Histopathological Evaluation

To evaluate histopathological changes in the liver, formalin‐fixed liver tissue samples were enclosed in paraffin, sliced using a microtome at 5 μm thickness, and mounted on regular microscope slides. Hematoxylin and eosin (H&E) staining was applied to the prepared slides, and the liver tissue samples were examined by a veterinary pathologist in a blinded manner.

### 2.4 Biochemical Analysis

#### 2.4.1 Measurement of Serum Alanine Aminotransferase (ALT) and Aspartate Aminotransferase (AST) Activities

Serum ALT and AST activities were determined in all groups using an autoanalyzer (Olympus AU‐600, Japan) and commercial kits (Pars Azmoon Company, Iran).

### 2.5 Evaluation of Serum Lipid Profile Levels

Serum levels of cholesterol (Chol), triglyceride (TG), and high‐density lipoprotein cholesterol (HDL‐C) were determined in all groups using commercial kits (Pars Azmoon Company, Iran) and an Olympus autoanalyzer (Olympus AU‐600, Japan). The concentration of low‐density lipoprotein cholesterol (LDL‐C) was estimated using the Friedewald equation (Friedewald et al. 1972).

### 2.6 Evaluation of Oxidative Stress Biomarkers

#### 2.6.1 Malondialdehyde (MDA) Concentration

The Nalondi‐Lipid Peroxidation Assay Kit (CAT NO: NS‐15022, Navand Salamat Co., Iran) was used to determine lipid peroxidation (LPO) in liver tissue samples. This assay is based on the combination of malondialdehyde (MDA), an LPO product, with 2‐thiobarbituric acid (TBA) to produce a pink solution (Dadpisheh et al. 2020). The absorbance of the colored solution was measured spectrophotometrically at 532 nm using an ELISA reader (Stat Fax 4700, USA).

#### 2.6.2 Paraoxonase 1 (PON1) Activity

Serum paraoxonase 1 (PON1) activity was measured using a Taligene Paraoxonase 1 Activity Assay Kit (CAT NO: TGP‐PON‐2011 96 T; Taligene Pars Co., Iran). Assessment of PON1 activity was based on the degradation of paraoxon to p‐nitrophenol, which is catalyzed by PON1 (Bełtowski et al. 2003; Dadpisheh et al. 2020). P‐nitrophenol generation was detected spectrophotometrically by measuring the increase in the absorbance at 412 nm.

### 2.7 Reverse Transcriptase‐Quantitative Polymerase Chain Reaction (RT‐qPCR)

The expression levels of RAS biomarkers (renin, angiotensinogen, AT1R, and ACE2) and inflammatory biomarkers (IL‐1β, IL‐6, TNF‐α, and IL‐10) were determined in the liver tissues using RT‐qPCR. Total RNA was extracted from frozen samples in liquid nitrogen using an RNA extraction kit (Yekta Tajhiz Azma, Iran), and complementary DNA (cDNA) was generated using a commercial cDNA synthesis kit (Yekta Tajhiz Azma, Iran). Genomic DNA was degraded from extracted RNA samples using DNase I (Yekta Tajhiz Azma, Iran). The desired gene expression levels were determined using RT‐qPCR and the BioFACT 2× real‐time PCR Master Mix (Biofact, Korea). β‐actin was used as an internal reference gene, and data analysis was performed using the 2^−ΔΔ*Cq*^ method. The primer sequences used for all reactions are listed in Table 1. RT‐qPCR reactions were replicated three times using an ABI instrument (7500, Applied Biosystems, USA). The following thermal cycling conditions were used for cDNA amplification: pre‐cycling heat activation at 95°C for 15 min (one cycle), denaturation at 95°C for 0.5 min (40 cycles), and annealing and extension at 95°C for 1 min (40 cycles).

**TABLE 1:** Primers selected for RT‐qPCR.

Gene	Forward primer (5′ → 3′)	Reverse primer (5′ → 3′)
IL‐10	ACCTCGTTTGTACCTCTCTCC	AGGAAGAACCCCTCCCATCAT
IL‐6	TCCTCTGGTCTTCTGGAGTAC	TCTGAAGGACTCTGGCTTTGT
TNF‐α	TAGCCCACGTCGTAGCAAAC	TAGACAAGGTACAACCCATCG
IL‐1β	TACAAGGAGAACCAAGCAACG	TCTTTCATTACACAGGACAGG
Renin	TTGTGAACTGTAGCCAGGTGC	TAGTCCGTACTGCTGAGTGTG
Angiotensinogen	CATCCTCCTCGAACTCAAAGC	TCGTAGATGGCGAACAGGAAG
AT1R	AACTCACAGCAACCCTCCAAG	TGCTTTTCTGGGTTGAGTTGG
ACE2	ACCCTTCCTACATCAGCCCCACTG	TGTCCAAAATCTACCCCACATAT
β‐Actin	GGATGCAGAAGGAGATTACTGC	CCACCGATCCACACAGAGTA

### 2.8 Western Blotting

A lysis buffer enriched with a protease inhibitor and Ripa lysate solution (Santa Cruz, USA) was used to lyse liver tissues. The samples were then centrifuged in an Eppendorf centrifuge at 12000 rpm for10 min at 4°C. The protein‐containing supernatant was extracted, and the protein content was measured using the Bradford technique, with absorbance measured at a wavelength of 630 nm. Protein samples were prepared using a solution of sodium dodecyl sulfate, and the protein molecules were separated based on their size using a technique known as gel electrophoresis. Following separation, the proteins were transferred from the gel onto a blotting membrane. Subsequently, the membrane was treated with a skim milk solution to prevent nonspecific binding. The membrane was subjected to incubation with the primary antibody (anti‐ACE2: sc‐73668, anti‐Ang1‐7: MBS2085958, Anti‐Actin: sc‐517582), followed by washing and subsequent incubation with the secondary antibody (anti‐mouse IgG‐HRP: ABIN3041713). The target protein band was detected using a chemiluminescence kit (Amersham, USA), and the film was visualized in a dark room using appearance and proofing solutions. After the appearance of the protein bands, ImageJ software (NIH, Bethesda, USA) was used for analysis.

### 2.9 Statistical Analysis

GraphPad Prism software (version 9.0.2.; San Diego, CA, USA) was used to evaluate the findings. Results were shown as the mean ± standard deviation (SD). Data distribution was determined using the one‐sample Kolmogorov–Smirnov test. One‐way ANOVA and Tukey's post hoc tests were used to determine statistical differences between the different groups. Sample size was determined using a power of 98%. The level of statistical significance was adjusted to *p* < 0.05.

## 3 Results

### 3.1 Effects of RSV With or Without AT on Liver Histopathological Changes in HFD‐Fed Mice

**FIGURE 1:**
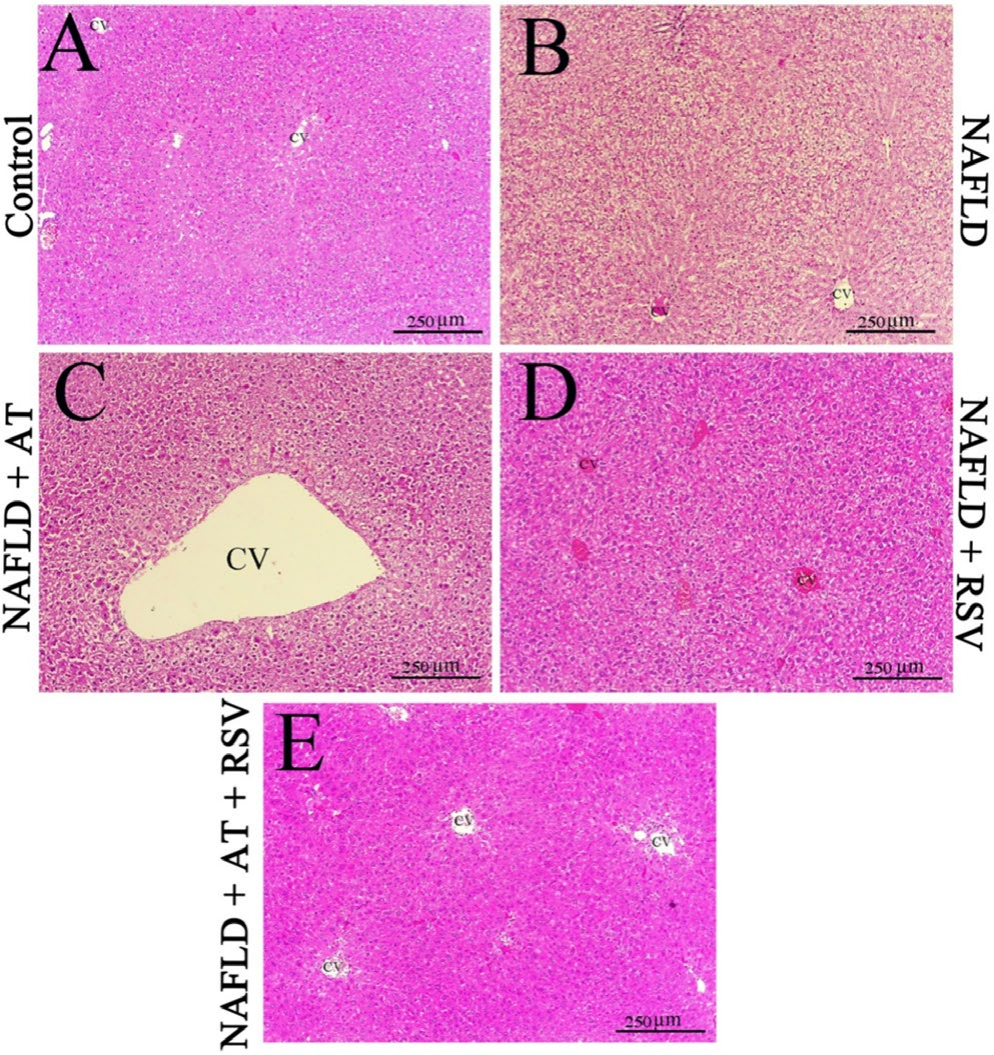
Effects of resveratrol (RSV) with or without atorvastatin (AT) on high‐fat diet (HFD)‐induced histopathological changes in mice (A–E). Control group (A); the hepatocytes are in a normal condition. Nonalcoholic fatty liver disease (NAFLD) group (B); uniformly, hepatocytes are swollen, and there are numerous fat‐containing vacuoles. Most nuclei are inclined from the center to the periphery of the cells. NAFLD + AT group (C); fatty cells are concentrated around the central vein (CV). However, the hepatocytes in the midzonal area are much less affected. NAFLD + RSV group (D); hepatocytes show infrequent changes. The nuclei are located at the center and only a rim of pale cream‐colored cytoplasm is prominent at the periphery of few hepatocytes. NAFLD + AT + RSV group (E); surprisingly, hepatocytes are in a normal condition with pink cytoplasmic staining and no evidence of fatty degeneration. CV, central vein.

H&E‐stained liver sections from mice in the control group showed a normal liver structure, in which hepatocytes were in a normal condition (Figure 1A), whereas 12 weeks of HFD administration led to histopathological changes in the liver, indicating the development of NAFLD. In HFD‐fed mice, hepatocytes were swollen and displayed a large number of fat‐containing vacuoles and nuclei disposed to the cell margin (Figure 1B). Conversely, hepatocytes from HFD‐fed mice treated with AT (20 mg/kg/day) in the midzonal area were much less affected. Fatty cells were concentrated around the central vein (CV) in liver sections from the NAFLD + AT group (Figure 1C). Interestingly, the NAFLD + RSV group exhibited hepatocytes with infrequent alterations compared with those in the NAFLD group (Figure 1D). In NAFLD mice treated with RSV, the nuclei were centrally located, with only a rim of pale creamy cytoplasm at the margins of a few hepatocytes. Intriguingly, treatment with RSV + AT considerably restored HFD‐induced histopathological changes compared with those in the NAFLD group, as manifested by normal hepatocytes with pink‐colored cytoplasm and no fatty degeneration (Figure 1E).

### 3.2 Effects of RSV With or Without AT on Serum ALT and AST Activities in HFD‐Fed Mice

**FIGURE 2:**
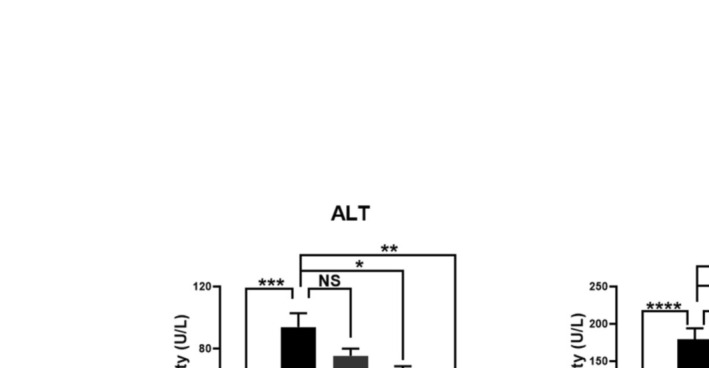
Effects of resveratrol (RSV) with or without atorvastatin (AT) on serum alanine aminotransferase (ALT) and aspartate aminotransferase (AST) activities. Bars represent mean ± standard deviation (SD). *p < 0.05, **p < 0.01, ***p < 0.001, and ****p < 0.0001. NS, not significant.

After 12 weeks of HFD administration, the serum activities of ALT and AST were significantly higher (1.78‐fold and 4.06‐fold, respectively) in the NAFLD group than in the control group (*p* = 0.0007 and < 0.0001, respectively; Figure 2). Treatment of NAFLD mice with AT (20 mg/kg) led to a slight decrease in serum ALT and AST activities (19.87% and 23.25%, respectively) compared with untreated NAFLD mice; however, these changes were not statistically significant (*p* > 0.05; Figure 2). In contrast, the administration of RSV (100 mg/kg) caused a significant decrease (29.83% and 34.69%, respectively) in serum ALT and AST activities in the NAFLD + RSV group compared to those in the NAFLD group (*p* = 0.01 and 0.02, respectively; Figure 2). NAFLD mice treated with both AT and RSV exhibited a significant decrease (39.72% and 40.04%, respectively) in the serum activities of ALT and AST compared with untreated NAFLD mice (*p* = 0.001 and 0.006, respectively).

### 3.3 Effects of RSV With or Without AT on Serum Lipid Profile Levels in HFD‐Fed Mice

Serum lipid profiles in the various groups are shown in Table 2. NAFLD mice had higher Chol, TG, and LDL‐C levels (2.38‐, 2.28‐, and 12.34‐fold, respectively) than healthy mice (*p* < 0.0001, < 0.0001, and < 0.0001, respectively). In contrast, HDL‐C levels were significantly reduced (1.57‐fold) in NAFLD mice compared with those in healthy mice (*p* = 0.003). Treatment of NAFLD mice with AT (20 mg/kg) significantly decreased the serum concentrations of Chol, TG, and LDL‐C (38.59%, 33.77%, and 67.94%, respectively) compared with untreated NAFLD mice (*p* = 0.0001, 0.001, and < 0.0001, respectively). However, AT treatment increased HDL‐C concentration (54.98%) in the NAFLD + AT group compared with that in the NAFLD group (*p* = 0.004). Additionally, RSV‐treated NAFLD mice showed significantly lower levels of Chol, TG, and LDL‐C (28.43%, 27.04%, and 48.62%, respectively) and significantly higher levels of HDL‐C (39.67%) than untreated NAFLD mice (*p* = 0.002, 0.007, 0.002, and 0.04, respectively). Treatment with AT and RSV significantly reduced serum concentrations of Chol, TG, and LDL‐C (37.36%, 31.74%, and 64.44%, respectively) and significantly increased serum HDL‐C concentrations (46.70%) in the NAFLD + AT + RSV group compared to the NAFLD group (*p* = 0.0001, 0.002, 0.0001, and 0.01, respectively).

**TABLE 2:** Effects of RSV with or without AT on serum lipid profile levels in HFD‐fed mice.

Group	Cho (mg/dL)	TG (mg/dL)	HDL‐C (mg/dL)	LDL‐C (mg/dL)
Control	59.6 ± 6.7	95.0 ± 16.8	34.4 ± 5.1	6.2 ± 3.3
NAFLD	142.1 ± 14.3*	216.7 ± 24.0*	21.8 ± 2.4*	76.8 ± 11.1*
NAFLD + AT	87.2 ± 6.6* ^,^ ^#^	143.5 ± 17.7* ^,^ ^#^	33.9 ± 4.4^#^	24.6 ± 3.9^#^
NAFLD + RSV	101.7 ± 17.2* ^,^ ^#^	158.1 ± 23.8* ^,^ ^#^	30.5 ± 4.2^#^	39.4 ± 16.1* ^,^ ^#^
NAFLD + AT + RSV	89.0 ± 11.9* ^,^ ^#^	147.9 ± 18.4* ^,^ ^#^	32.1 ± 2.5^#^	27.3 ± 14.5^#^

*Note:* All of the values are represented as mean ± standard deviation (SD).

Abbreviations: AT, atorvastatin; Cho, cholesterol; HDL‐C, high‐density lipoprotein cholesterol; LDL‐C: low‐density lipoprotein cholesterol; NAFLD, nonalcoholic fatty liver disease; RSV, resveratrol; TG, triglyceride.

* Statistically significant difference compared with the control group at *p* < 0.05.

^#^ Statistically significant difference compared with the NAFLD group at *p* < 0.05.

### 3.4 Effects of RSV With or Without AT on Hepatic Oxidative Stress in HFD‐Fed Mice

**FIGURE 3:**
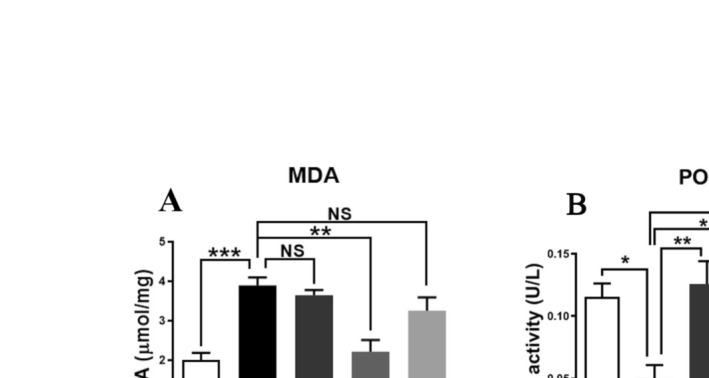
Effects of resveratrol (RSV) with or without atorvastatin (AT) on liver malondialdehyde (MDA) level and serum paraoxonase 1 (PON1) activity in high‐fat diet (HFD)‐fed mice. Bars represent mean ± standard deviation (SD). *p < 0.05, **p < 0.01, and ***p < 0.001. NS, not significant.

The status of liver oxidative stress biomarkers, MDA and PON1, in the different groups is presented in Figure 3. As expected, there was a significant increase in liver MDA levels (1.94‐fold) in the NAFLD group compared with that in the control group (*p* = 0.0004; Figure 3A). Treatment with RSV (100 mg/kg) significantly decreased MDA levels (43.21%) in the RSV group compared with those in the NAFLD group (*p* = 0.001; Figure 3A); however, the decrease in MDA levels (6.51%) was not statistically significant after AT (20 mg/kg) treatment (*p* = 0.941; Figure 3A). Additionally, MDA levels were decreased (16.38%) in HFD‐fed animals that received both AT and RSV, but this difference was not statistically significant compared with the NAFLD group (*p* = 0.369; Figure 3A).

HFD administration for 12 weeks led to a considerable decrease in serum PON1 activity (2.31‐fold) in the NAFLD group compared with that in the control group (*p* = 0.011; Figure 3B). Conversely, serum PON1 activity was significantly enhanced in NAFLD mice (151.6% and 111%, respectively) following treatment with AT and RSV (*p* = 0.003 and 0.035, respectively; Figure 3B). Furthermore, mice with NAFLD treated with both AT and RSV showed a significant increase in serum PON1 activity (140%) compared with untreated NAFLD mice (*p* = 0.012; Figure 3B).

### 3.5 Effects of RSV With or Without AT on Inflammatory Biomarkers in HFD‐Fed Mice

**FIGURE 4:**
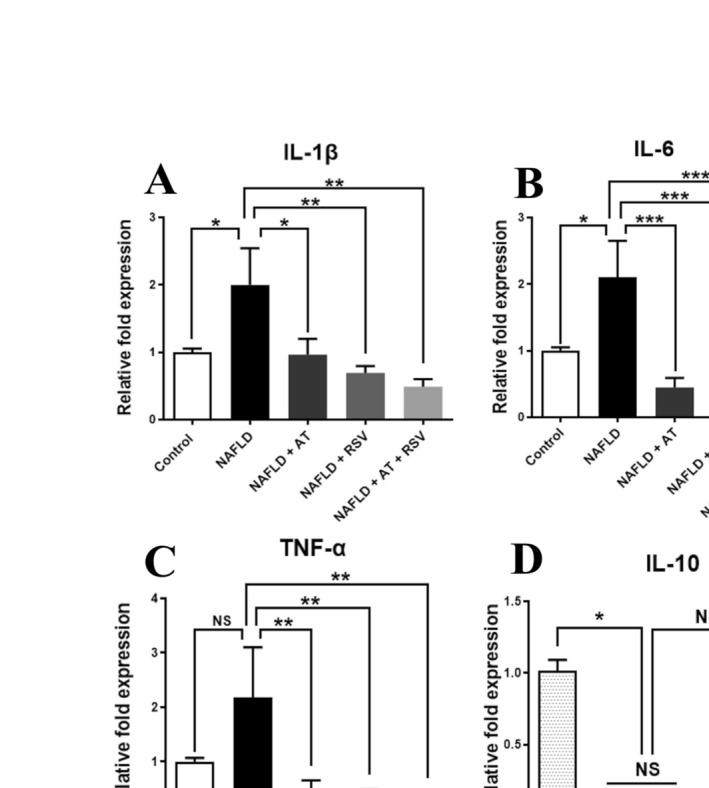
Effects of resveratrol (RSV) with or without atorvastatin (AT) on inflammatory biomarkers in high‐fat diet (HFD)‐fed mice. Bars show mean ± standard deviation (SD). *p < 0.05, **p < 0.01, ***p < 0.001, and ****p < 0.0001. NS, not significant.

To determine whether pro‐inflammatory and anti‐inflammatory cytokines are affected by RSV and AT, HFD‐fed animals were treated with AT, RSV, and AT + RSV, and the expression levels of these cytokines were assessed using RT‐qPCR. The expression levels of IL‐1β, IL‐6, and TNF‐α were markedly higher in the NAFLD group (2‐fold, 2.1‐fold, and 2.18‐fold, respectively) than in the control group (*p* = 0.03, 0.01, and 0.07, respectively; Figure 4A–C). In contrast, AT or RSV treatment significantly decreased the elevation of IL‐1β (*p* = 0.028 and 0.004, respectively), IL‐6 (*p* = 0.0003 and 0.0007, respectively), and TNF‐α (*p* = 0.006 and 0.003, respectively) gene expression in NAFLD mice (Figure 4A–C). Interestingly, the NAFLD + AT + RSV group exhibited a more pronounced decrease in IL‐1β, IL‐6, and TNF‐α expression (4.09‐fold, 26.90‐fold, and 5.47‐fold, respectively) than the other treatment groups (*p* = 0.001, < 0.0001, and 0.003, respectively; Figure 4A–C).

NAFLD mice showed significantly reduced IL‐10 expression (6.49‐fold) compared with healthy mice (*p* = 0.045; Figure 4D). Conversely, RSV alone or in combination with AT increased IL‐10 expression compared with that in the NAFLD group (3.69‐fold and 3.54‐fold, respectively), but these changes were not statistically significant (*p* = 0.595 and 0.644, respectively; Figure 4D). The expression levels of IL‐10 were not affected by AT in the HFD‐fed animals (*p* > 0.999; Figure 4D).

### 3.6 Effects of RSV With or Without AT on the Angiotensinogen/Renin/AT1R Axis in HFD‐Fed Mice

**FIGURE 5:**
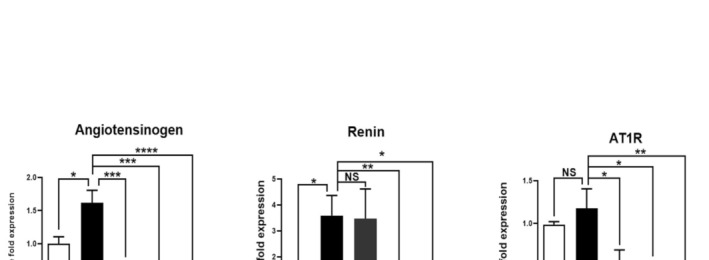
Effects of resveratrol (RSV) with or without atorvastatin (AT) on the expression of the classical renin‐angiotensin system (RAS) components in high‐fat diet (HFD)‐fed mice. Bars show mean ± standard deviation (SD). *p < 0.05, **p < 0.01, ***p < 0.001, and ****p < 0.0001. NS, not significant.

For the first time, we evaluated the effects of RSV with or without AT on the classical RAS axis by determining angiotensinogen, renin, and AT1R gene expression. As indicated in Figure 5, the expression levels of angiotensinogen, renin, and AT1R in the liver were remarkably increased (1.61‐fold, 3.59‐fold, and 1.17‐fold, respectively) after 12 weeks of HFD administration (*p* = 0.043, 0.048, and 0.879, respectively). AT treatment resulted in a significant decrease in angiotensinogen and AT1R expression (68.31% and 56.51%, respectively) compared with the NAFLD group (*p* = 0.0003 and 0.028, respectively). However, renin expression was not significantly altered in AT‐treated NAFLD mice (*p* > 0.9999). Interestingly, 4 weeks of RSV administration led to a marked decrease in angiotensinogen, renin, and AT1R expression (75.01%, 97.98%, and 63.22%, respectively) compared to that in the NAFLD group (*p* = 0.0002, 0.006, and 0.012, respectively; Figure 5). Administration of RSV enhanced the inhibitory effects of AT on the classical RAS axis, as manifested by a remarkable decrease in angiotensinogen, renin, and AT1R expression (91.08%, 82.16%, and 71.71%, respectively) in the NAFLD + AT + RSV group compared to those in the NAFLD group (*p* < 0.0001, 0.032, and 0.004, respectively; Figure 5).

### 3.7 Effects of RSV With or Without AT on the ACE2/Ang1‐7 Axis in HFD‐Fed Mice

**FIGURE 6:**
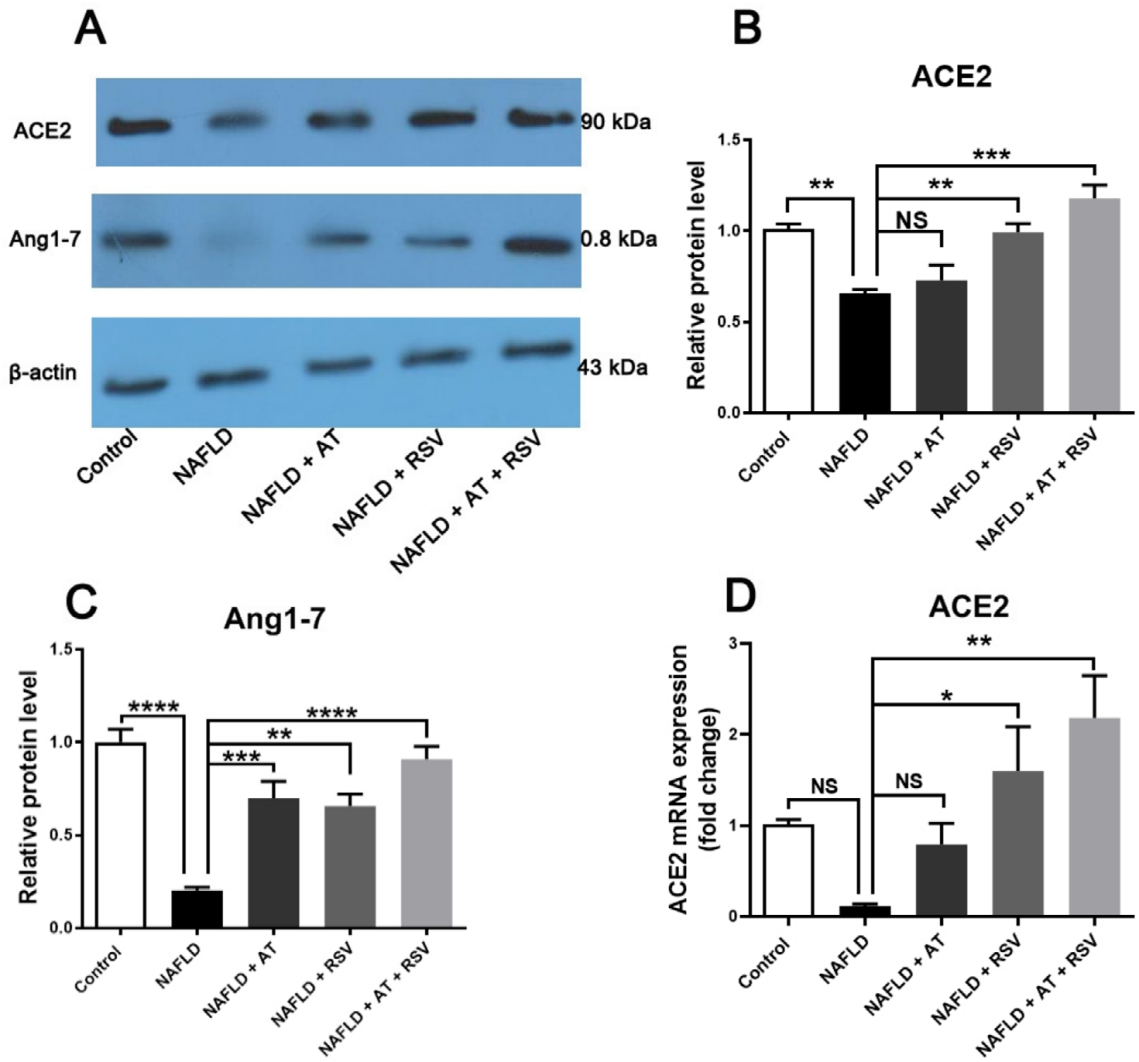
Effects of resveratrol (RSV) with or without atorvastatin (AT) on the expression of the alternative renin‐angiotensin system (RAS) components in high‐fat diet (HFD)‐fed mice. Bars show mean ± standard deviation (SD). *p < 0.05, **p < 0.01, ***p < 0.001, and ****p < 0.0001. NS, not significant.

To evaluate the effects of RSV and AT on the nonclassical RAS axis, we determined the expression levels of ACE2 and Ang1‐7 in all studied groups. As indicated in Figure 6, ACE2 and Ang1‐7 protein expression levels were lower (1.52‐fold and 4.93‐fold, respectively) in the NAFLD group than in the control group (*p* = 0.009 and < 0.0001, respectively). However, the decrease in ACE2 mRNA expression (8.96‐fold) was not statistically significant in the NAFLD group compared to that of the control group (*p* = 0.393; Figure 6D). Treatment with AT significantly enhanced Ang1‐7 protein expression (3.45‐fold) in AT‐treated NAFLD mice compared with that in untreated NAFLD mice (*p* = 0.0007; Figure 6). However, AT did not lead to a significant alteration in ACE2 protein and mRNA expression in NAFLD mice (*p* > 0.05). In contrast, RSV‐treated NAFLD mice showed a remarkable increase in ACE2 protein and mRNA expression (*p* = 0.008 and 0.04, respectively) and Ang1‐7 protein expression (*p* = 0.001) compared with untreated NAFLD mice. Interestingly, ACE2 protein and mRNA expression and Ang1‐7 protein expression were significantly upregulated in NAFLD mice after co‐treatment with AT and RSV (*p* = 0.0001, 0.003, and < 0.0001, respectively; Figure 6). As expected, the upregulation of ACE2 and Ang1‐7 was more pronounced in NAFLD mice after the co‐administration of RSV and AT (Figure 6).

## 4 Discussion

The current study aimed to investigate the effects of RSV with or without AT on liver histopathological changes, oxidative stress, and classical and nonclassical RAS axes in mice with NAFLD. Our findings demonstrated that 4 weeks of treatment of NAFLD mice with RSV and AT could regenerate most of the altered parameters after 12 weeks of HFD administration. Interestingly, these regenerative effects were more pronounced in NAFLD animals that received RSV and AT than in those that received RSV or AT alone.

The administration of HFD has been widely accepted for the development of NAFLD in experimental animals. Consistent with former studies (Azeemuddin et al. 2016; Reda et al. 2023), HFD administration for 12 weeks effectively increased TG, Chol, and LDL‐C levels, and decreased HDL‐C levels. Additionally, HFD mice developed hepatic steatosis, as indicated by swollen hepatocytes with a large number of fat‐containing vacuoles and nuclei predisposed to cell margins. Intrahepatic fat accumulation led to a marked elevation in serum ALT and AST activities, confirming hepatocellular injury. However, the histopathological findings in NAFLD mice treated with AT or RSV were quite different, with the greatest regenerative effects observed in AT + RSV‐treated NAFLD mice. Normal hepatocytes with a pink cytoplasm and no signs of fatty degeneration were observed in this group. In addition, the NAFLD + AT + RSV group showed ameliorated dyslipidemia and decreased serum ALT and AST activities, indicating the hepatoprotective and hypolipidemic effects of AT and RSV. These regenerative effects could be attributed to enhanced fatty acid oxidation and decreased oxidative stress and inflammation following RSV and AT treatments (Park et al. 2016; Zamani et al. 2017; Gimeno‐Mallench et al. 2019).

Oxidative stress is a key mechanism underlying NAFLD pathogenesis (Delli Bovi et al. 2021). Parallel to previous studies (Attia et al. 2022; Bae et al. 2024), NAFLD development in mice was accompanied by enhanced oxidative stress, as manifested by elevated MDA levels and reduced PON1 activity. Our results demonstrated that 4 weeks of RSV administration with or without AT enhanced PON1 activity. However, a significant decrease in MDA concentration was observed only in NAFLD mice that received RSV. Our findings are in accordance with those of previous studies that reported the antioxidant activities of RSV and AT (Ara et al. 2005; Zhang et al. 2015). These antioxidant properties may be associated with the ability of RSV and AT to scavenge free radicals (Lee et al. 2016; Gu et al. 2021). Additionally, increased PON1 activity may be explained by the potential of RSV and AT to increase PON1 gene expression in hepatocytes. Interestingly, RSV has been reported to activate the aryl hydrocarbon receptor (AhR), which in turn binds to an unusual AhR‐responsive element within the promoter region of PON1, resulting in its upregulation (Gouédard et al. 2004). Statins have been shown to enhance PON1 promoter activity and subsequently its expression by activating the transcription factors Sp1 and SREBP‐2 (Costa et al. 2011). Therefore, the amelioration of oxidative stress by RSV and AT may be related to their ability to scavenge ROS and/or induce PON1 gene expression.

Consistent with previous studies (Wu et al. 2019; McCall et al. 2023), our findings indicated aberrant expression of the pro‐inflammatory cytokines IL‐1β, IL‐6, and TNF‐α, as well as decreased expression of the anti‐inflammatory cytokine IL‐10 in NAFLD mice. This inflammatory response is triggered mainly by the NF‐κB pathway in NAFLD, in which p65 is translocated from the cytoplasm to the nucleus and induces the expression of pro‐inflammatory cytokines (Sangouni et al. 2019; Zhao et al. 2022). IL‐10 downregulation is associated with T‐helper 2 (Th2) lymphocyte dysfunction (Cano Barquilla et al. 2014). In contrast, treatment of NAFLD mice with RSV, AT, and RSV + AT considerably modulated pro‐inflammatory cytokines, with more pronounced anti‐inflammatory effects in RSV + AT‐treated NAFLD mice. The reduction in pro‐inflammatory cytokine expression after RSV treatment may be explained by the potential of RSV to suppress the NF‐κB pathway by decreasing p65 subunit expression and inhibiting IκB protein degradation through suppressing IκB kinases (IKKs) (PMID: 24020126). In line with previous reports (Tao et al. 2016), treatment with RSV and RSV + AT remarkably increased liver IL‐10 expression, which may be the result of promoting the differentiation of Th2 cells that release anti‐inflammatory cytokines (Wang et al. 2014). This hypothesis needs to be further elucidated in NAFLD mice.

It is well known that an imbalance between the RAS axes arises in NAFLD, wherein Ang II exhibits greater effects than Ang1‐7 (Cao et al. 2016; Wu et al. 2016). Accumulating evidence has shown that dysregulation of the classical RAS pathway increases oxidative stress and inflammation, contributing to liver injury and fibrosis in patients with NAFLD. For example, Goh et al. (2015) reported that patients with NAFLD who received classical RAS blockers exhibited lower levels of liver fibrosis. Conversely, the activation of the nonclassical pathway has anti‐inflammatory and antioxidant effects, suggesting potential therapeutic benefits (Cao et al. 2016). In this study, 12 weeks of HFD administration activated the classical RAS axis, as evidenced by the marked upregulation of angiotensinogen, renin, and AT1R. In agreement with previous studies (Romão et al. 2020; Yang et al. 2020), activation of the classical RAS axis was accompanied by increased oxidative stress, inflammation, and fatty liver degeneration in NAFLD mice. Conversely, ACE2 and Ang1‐7 were considerably downregulated following NAFLD development, indicating the suppression of the alternative RAS axis. Treatment of NAFLD mice with RSV and RSV + AT considerably modulated the RAS balance. Interestingly, RSV + AT exhibited greater regulatory effects on the RAS axes than those mediated by AT or RSV alone. These findings are aligned with previous reports showing that blocking the classical RAS axis and activating the ACE2/Ang1‐7 axis elicited beneficial effects in NAFLD (Takahashi et al. 2007; Jayasooriya et al. 2008; Cao et al. 2016; Attia et al. 2022). Enhanced expression of Ang1‐7 has been demonstrated to decrease gluconeogenesis, lipogenesis, oxidative stress, and inflammation while promoting fatty acid oxidation (Yang et al. 2020; Attia et al. 2022). Therefore, regulation of the RAS axes seems to be essential for RSV‐ and AT‐mediated attenuation of NAFLD. The PI3K/AKT pathway has been reported to be essential for regulating RAS axes (Yang et al. 2020). Interestingly, Shu et al. indicated that RSV upregulated PI3K and Akt expression in HFD‐fed mice (Shu et al. 2020). Additionally, AT has been identified as a trigger for the PI3K/AKT pathway (Zhang et al. 2007). Altogether, the modulation of the RAS axes by AT and RSV may be mediated by PI3K/AKT pathway activation, which requires further exploration in NAFLD mice.

Our results, which indicate the regulation of classical and nonclassical RAS axes by AT and RSV, highlight their potential to alter RAS balance in favor of a protective phenotype. Targeting the RAS pathway with RSV and statins may not only improve liver pathology but also ameliorate systemic outcomes, such as cardiovascular and metabolic health, which are common comorbidities in patients with NAFLD (Glass et al. 2019; Hou et al. 2019). Therefore, the regulation of RAS presents a promising avenue for integrated treatment strategies in clinical settings. The translational potential of these findings lies in their applicability in clinical interventions.

## 5 Conclusions

This study elucidated that RSV ameliorated oxidative stress and restored fatty liver degeneration in mice with NAFLD. It also relieved inflammation by suppressing pro‐inflammatory cytokines and upregulating IL‐10. Additionally, RSV modulated the RAS axes, suppressing the angiotensinogen/renin/AT1R axis and activating the ACE2/Ang1‐7 axis. Interestingly, these beneficial effects were comparable to those of AT and were more pronounced when RSV and AT were co‐administered. Overall, the beneficial effects of RSV and AT may be at least partially mediated by the regulation of the RAS axes, which present a new window into the mechanisms of RSV and AT in NAFLD treatment.

## Author Contributions

**Esmaeel Babaeenezhad:** conceptualization (equal), investigation (equal), methodology (equal), writing – original draft (equal), writing – review and editing (equal). **Navid Farahmandian:** conceptualization (equal), investigation (equal), methodology (equal). **Mohammadjavad Sotoudeheian:** investigation (equal), methodology (equal), software (equal), writing – original draft (equal). **Omid Dezfoulian:** investigation (equal), methodology (equal), software (supporting). **Elaheh Askari:** investigation (equal), methodology (equal), writing – original draft (equal). **Niloofar Taghipour:** conceptualization (equal), investigation (equal), methodology (equal), writing – original draft (equal). **Sahar Yarahmadi:** conceptualization (equal), investigation (equal), methodology (equal), writing – original draft (equal), writing – review and editing (equal).

## Ethics Statement

All animal experiments were conducted in accordance with the principles of the Declaration of Helsinki. Animal experiments were performed in accordance with the ethical guidelines and regulations set by the Ministry of Health of Iran (ethical code: IR.SBMU.AEC.1402.058, Research Number: 43005377).

## Consent

The authors have nothing to report.

## Conflicts of Interest

The authors declare no conflicts of interest.

## Data Availability

The authors have nothing to report.
